# Patient Outcomes After Anterior Cruciate Ligament (ACL) Reconstruction With Hybrid Allograft Augmentation Compared With Isolated Autografts: A Systematic Review and Meta-Analysis

**DOI:** 10.7759/cureus.114231

**Published:** 2026-08-09

**Authors:** Charlotte Wahle, Teana Tee, Alexis Cheney, Nicole J Newman-Hung, Andy Contreras, Dhruv Limaye, Thomas J Kremen

**Affiliations:** 1 Orthopedic Surgery, University of California, Los Angeles, USA; 2 Orthopedics, Herbert Wertheim College of Medicine, Florida International University, Miami, USA; 3 Department of Surgery, University of Washington, Seattle, USA; 4 Orthopedic Surgery, UCLA Medical Center, Los Angeles, USA

**Keywords:** allograft, anterior cruciate ligament, anterior cruciate ligament reconstruction, hamstring autograft, hybrid graft

## Abstract

Anterior cruciate ligament reconstruction (ACLR) is a common orthopedic procedure involving the replacement of a torn anterior cruciate ligament (ACL) with a graft, and current literature comparing the outcomes of different graft types remains inconclusive. The aim of this study was to compare isolated autografts and hybrid allograft/autograft grafts of various sizes to determine whether allograft augmentation provides a clinically significant benefit during ACLR by assessing patient-reported outcome measures (PROMs) and rates of graft failure. A comprehensive systematic review and meta-analysis was performed, with the search conducted across three databases (PubMed, Embase, and Cochrane Reviews). Quantitative data were collected regarding PROMs and rates of re-tear/revision, and a meta-analysis was performed to assess Lysholm, International Knee Documentation Committee (IKDC), and Tegner scores and rates of graft failure. The initial search yielded 249 studies, of which 17 met the final inclusion criteria; the included studies were published between 2015 and 2025, and 15 of the 17 were retrospective cohort studies. In terms of pooled patient outcomes, there were no clinically significant differences between hybrid grafts and isolated autografts across any of the analyzed clinical scores. Lysholm scores were statistically higher in the autograft group (weighted mean difference (WMD) 3.77, 95% CI 1.47-6.07, p=0.0013), though this difference remained below the established minimal clinically important difference; IKDC (p=0.80) and Tegner (p=0.28) scores did not differ significantly between groups. The pooled relative risk of graft failure was slightly higher in patients with hybrid grafts (risk ratio (RR) 1.24, 95% CI 0.84-1.84); however, this difference was not statistically significant (p=0.28). At the individual study level, there is literature to both support and refute the viability of hybrid ACL grafts in augmenting an autograft of insufficient diameter. However, following a comprehensive review of the literature and meta-analysis, we report a lack of clinically meaningful or statistically significant differences in clinical outcome scores and overall rates of graft failure.

## Introduction and background

Anterior cruciate ligament reconstruction (ACLR) is a common orthopedic procedure, with more than 200,000 surgeries performed per year in the United States [1-7]. The anterior cruciate ligament (ACL) is the primary stabilizing structure preventing anterior translation and rotational instability of the tibia relative to the femur, and when torn, it is typically reconstructed using donor tissue (graft) rather than repaired directly, given the ligament's limited intrinsic healing capacity. Different types of grafts can be utilized for ACLR, such as autografts, allografts, and hybrid grafts [8-11]. Autografts and allografts can be harvested from various locations in the lower extremity, including the patellar tendon, quadriceps tendon, hamstring tendon, or other soft tissues [8-10,12-14]. Some factors in graft selection depend on intrinsic patient factors (e.g., patient age, sex, activity level, and body habitus), while other aspects are more a function of surgeon preference based on training, procedural comfort, graft site morbidity, and prior observed outcomes in their practice [4].

As graft harvesting can be unpredictable, graft size may ultimately be smaller than expected, raising the question of its sufficiency for ACLR. Although no universally accepted cutoff exists, graft diameters below approximately 7-8 mm are commonly considered 'small' and have been associated with increased rates of graft failure in multiple cohort studies, while diameters of 8 mm or greater are generally considered 'sufficient' [15,16]. Hybrid graft augmentation is one approach when the harvested autograft in isolation may be too small in diameter [15,16]. This method involves combining an autograft from the patient with an allograft from a cadaver [15-17]. The primary aim of the hybrid graft is to improve the tensile strength and durability of the graft to effectively reduce the retear rate; however, whether hybrid grafts truly deliver the intended result remains unclear [17].

Previous studies have attempted to analyze the clinical outcomes of hybrid grafts compared with isolated grafts but have yielded mixed results [9,17-19]. Despite the arguments of proponents of hybrid grafts, numerous studies have reported no correlation between autograft augmentation and retear rate [9,10,15,20,21]. In contrast, several studies argue that hybrid grafts remain inferior to isolated autografts regardless of graft diameter. Hybrid constructs tend to exhibit the biological characteristics of allografts, showing delayed revascularization that may negate the mechanical benefits of a larger graft [19,22-24]. The present study evaluated clinical outcomes and graft failure rates following ACLR with hybrid grafts versus isolated autografts. Failure rates were defined as graft rupture and/or the need for ACLR revision. The findings may help guide decision-making regarding the use of hybrid graft augmentation.

## Review

Materials and methods

A systematic review was conducted in accordance with the Preferred Reporting Items for Systematic Reviews and Meta-Analyses guidelines. The search was conducted on July 25, 2024, across the databases PubMed, Embase, and Cochrane Reviews using Boolean search phrases: (“allograft augmentation” OR “allograft supplementation” OR “graft augmentation” OR “tissue augmentation” OR “hybrid graft”) AND (“ACL reconstruction” OR “anterior cruciate ligament reconstruction” OR “ACL repair” OR “anterior cruciate ligament repair”). The search was rerun on June 6, 2025, to capture literature published after the original search date. Inclusion criteria consisted of all original research studies published in English that compared outcomes between patients undergoing hybrid autograft/allograft ACLR and patients undergoing isolated autograft ACLR with a minimum of one year of follow-up. Exclusion criteria consisted of studies not published in English, studies without documented clinical patient outcomes for each group (simulation-based or cadaveric studies), studies without full-text availability, and nonoriginal research (systematic reviews/meta-analyses).

Studies were initially screened based on title and abstract, after which they were read in full to evaluate whether they met the inclusion and exclusion criteria. References cited in each included study were screened for additional relevant research that had been missed in the database search. A reverse search was also conducted to identify recent papers that had cited the included studies as references. While review papers were excluded as a category, reviews and meta-analyses were screened for references to relevant original research. Each study was independently evaluated by at least two authors, and the inclusion and exclusion criteria were strictly applied using a predefined protocol. Discrepancies were resolved by a board-certified sports medicine orthopedic surgeon.

Data extraction considered the following: key characteristics of each included study (author, publication date, study design, patient demographics, and sample size), details on the intervention, and patient outcomes (patient-reported outcome measures (PROMs) and graft failure). Patient demographics included age range and mean. Intervention details comprised graft type, augmentation type, and graft diameter, if available. Graft failure was defined by pathologic laxity (inability to perform activities of daily living after previously being able to do so, positive Lachman test, positive pivot-shift test, or KT-1000 side-to-side difference greater than 3 mm), rupture confirmed on imaging, or the need for reoperation/revision surgery. Minimal clinically important difference (MCID) was defined per existing literature [25-27].

The three validated PROMs that were included were the Lysholm knee score, International Knee Documentation Committee (IKDC) score, and Tegner activity scale. The Lysholm knee score is a summation of eight items encompassing knee pain, functionality, and stability. It is assessed on a scale of 0 to 100, with higher scores indicating a lower degree of knee instability (i.e., better function) [28,29]. The IKDC score is also a measure of knee stability, covering knee symptoms (pain, edema, and stiffness), performance in activity, and functionality. It is measured on a scale of 0 to 100, with higher scores signifying a lower degree of symptoms (i.e., better function) [30]. The Tegner activity scale determines the activity levels of a patient prior to injury and post-surgery. It is measured on a scale of 0 to 10, with higher scores indicative of a higher level of activity (e.g., engagement in competitive sports) [31-33].

Statistical analysis

Statistical analysis was performed using basic descriptive statistics (mean, median, mode, and range) as well as meta-analysis where appropriate. Meta-analysis was carried out for the risk ratio of the retear/revision rate and for common clinical scores where at least three separate studies reported findings. For the retear/revision outcome, study-level risk ratios were calculated from raw event counts on the log scale; for continuous clinical scores (Lysholm, IKDC, and Tegner), study-level weighted mean differences (WMDs) were calculated between hybrid and autograft groups. Given substantial heterogeneity across studies (I² > 50% for three of four outcomes), all pooled estimates were generated using a DerSimonian-Laird random-effects model in response to the observed heterogeneity. Two-sided p-values, 95% confidence intervals, and the I² statistic were calculated for each pooled estimate. Publication bias was assessed qualitatively via funnel plots and quantitatively using Egger's regression test for each outcome. Results are interpreted cautiously for outcomes with fewer than 10 included studies, given the reduced power of this test in small meta-analyses. All statistical analyses were performed using Stata v18 (StataCorp, College Station, TX).

Risk of bias and methodological quality assessment

The methodological quality and risk of bias of the included studies were assessed using the Newcastle-Ottawa Scale (NOS) for nonrandomized studies and the Cochrane risk of bias (RoB 2) tool for randomized trials. The NOS evaluates studies across selection, comparability, and outcome domains, with a maximum score of nine stars. In this research, studies scoring 7-9 stars were considered high quality, 4-6 stars moderate quality, and 0-3 stars low quality. In addition, a randomized controlled trial (RCT) was assessed using the RoB 2 tool across its five standard domains. Disagreements in risk-of-bias scoring were resolved using the same adjudication process described above for study selection.

Results

**Figure 1:**
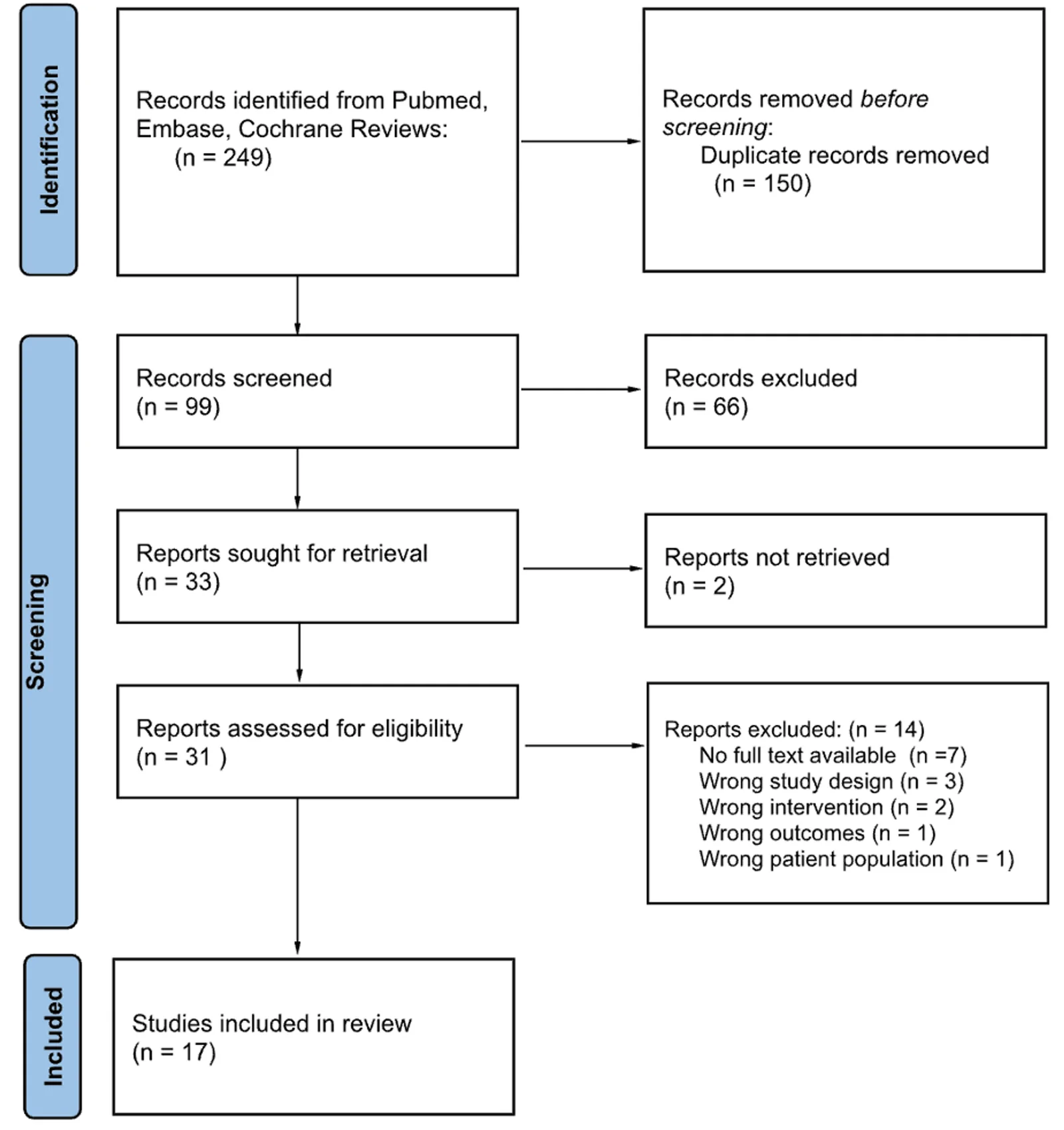
PRISMA flowchart PRISMA, Preferred Reporting Items for Systematic Reviews and Meta-Analyses

A total of 249 studies emerged from the initial search. After title and abstract screening, 99 studies remained. Figure 1 presents the search and selection process. Ultimately, 17 studies met the inclusion criteria, 15 (88%) of which were retrospective cohort studies [10,12,14,15,17-24,34-36]. One study was a prospective RCT [37]. One study was a nonrandomized prospective cohort study [38]. All 17 included studies compared objective measures and clinical outcomes in patients who underwent hybrid autograft/allograft ACLR versus isolated autograft reconstruction. The characteristics and primary findings, including study design, graft types, graft failure definitions, and key findings, are summarized in Table 1.

**Table 1:** Characteristics and primary findings of included studies Sample size reported as autograft (A), hybrid (H), or allograft (Allo); a dash indicates that the arm was not present. Clinical significance is defined in the Methods for each scoring system. ACLR, anterior cruciate ligament reconstruction; MRI, magnetic resonance imaging; ADLs, activities of daily living; KT-1000, arthrometer; IKDC, International Knee Documentation Committee; KOOS, Knee Injury and Osteoarthritis Outcome Score

First author, year	Study type	Sample size, n (A/H/allo)	Mean age (years)	Diameter threshold for augmentation	Graft types included	Graft failure definition	Key findings
Adams, 2023 [15]	Retrospective cohort	49/63/-	29.2	<8.0 mm	Semitendinosus, gracilis	Graft rupture confirmed on MRI and/or need for revision ACLR	Clinical scores: no difference. Graft failure: no difference.
Burrus, 2015 [17]	Retrospective cohort	29/29/-	27.2 (A), 26.6 (H)	<7.5 mm	Semitendinosus, gracilis	Revision ACLR or complete graft rupture on MRI	Clinical scores: better IKDC and Lysholm in autograft. Graft failure: higher in hybrid.
Darnley, 2016 [38]	Prospective cohort	27/27/-	20.9	<8.5 mm	Semitendinosus, gracilis	Revision ACL surgery	Clinical scores: no difference. Graft failure: no difference.
Heyworth, 2021 [20]	Retrospective cohort	64/47/-	15.8	Not specified	Semitendinosus, gracilis, tibialis anterior/posterior	Retear; revision (need for additional surgery)	Clinical scores: no difference. Graft failure: no difference.
Jacobs, 2017 [18]	Retrospective cohort	46/42/-	15	Not specified	Semitendinosus, gracilis	Patient-reported instability affecting ADLs/sport, pathological laxity on exam, or failed graft on MRI/arthroscopy	Clinical scores: not available. Graft failure: higher in autograft.
Kraeutler, 2018 [12]	Retrospective cohort	83/65/-	36	<7.5 mm (3/4 surgeons); <7.0 mm (1 surgeon)	Semitendinosus, gracilis	Revision rate (later revision ACLR/total primary ACLR)	Clinical scores: better IKDC in hybrid (not clinically significant). Graft failure: no difference.
Leo, 2016 [10]	Retrospective cohort	71/24/-	26.9 (A), 28.1 (H)	<8.0 mm	Semitendinosus, gracilis, tibialis	Retear by history and exam (Lachman, pivot shift), confirmed on MRI	Clinical scores: no difference. Graft failure: no difference.
Li, 2015 [37]	Randomized controlled trial	32/31/32	30	N/A (randomized a priori)	Semitendinosus, gracilis, tibialis anterior	Not measured	Clinical scores: no difference. Graft failure: not available.
Mirzayan, 2023 [21]	Retrospective cohort	1397/548/-	N/A	<8.0 mm	Tibialis anterior/posterior, semitendinosus, gracilis, Achilles, peroneus longus, or other	Aseptic revision (reoperation with original graft removed and replaced)	Clinical scores: no difference. Graft failure: no difference.
Pennock, 2017 [19]	Retrospective cohort	24/26/-	15.7	<7.0 mm	Semitendinosus, gracilis	Failure confirmed by exam and MRI, or revision ACLR for discontinuous graft confirmed at arthroscopy	Clinical scores: no difference. Graft failure: higher in hybrid.
Perkins, 2019 [22]	Retrospective cohort	289/65/-	15.3	Not a fixed threshold	Semitendinosus, gracilis, tibialis anterior	Revision ACLR within system, MRI evidence of complete rupture, or self-reported outside revision	Clinical scores: not available. Graft failure: higher in hybrid.
Rao, 2021 [34]	Retrospective cohort	80/59/-	22.9	<8.0 mm	Semitendinosus, gracilis	Revision rate (ACL failure); reoperation rate (recurrent ACL tear, meniscal pathology)	Clinical scores: no difference. Graft failure: no difference.
Wang, 2018 [23]	Retrospective cohort	29/28/-	32.6 (A), 32.8 (H)	<8.0 mm	Semitendinosus, gracilis	Patient-reported instability affecting ADLs/sport, pathologic laxity on exam (Lachman, pivot shift, KT-1000 >3 mm), or MRI	Clinical scores: better IKDC, Lysholm, and Tegner in autograft. Graft failure: no difference.
Xu, 2017 [24]	Retrospective cohort	31/37/-	32.8 (A), 33.9 (H)	Not a fixed mm threshold	Semitendinosus, gracilis	Revision ACL surgery	Clinical scores: better IKDC and Lysholm in autograft; lower Tegner in hybrid (not statistically significant). Graft failure: no difference.
Xu, 2018 [35]	Retrospective cohort	34/42/-	35	<8.5 mm	Semitendinosus, gracilis, tibialis anterior	Not measured	Clinical scores: better IKDC and Lysholm in autograft. Graft failure: not available.
Yang, 2025 [36]	Retrospective cohort	58/44/-	29.3 (A), 27.8 (H)	8.0 mm (fixed)	Semitendinosus, gracilis	Suspected on exam (Lachman, pivot shift), validated by MRI	Clinical scores: no difference. Graft failure: no difference.
Zheng, 2019 [14]	Retrospective cohort	28/32/37	30.6	<8.0 mm	Semitendinosus, gracilis, tibialis anterior	Not measured	Clinical scores: no difference. Graft failure: not available.

Risk of bias and methodological quality

Among the 16 nonrandomized studies, NOS scores ranged from 5 to 8 (median, 7), indicating moderate-to-high methodological quality (Table 2). Twelve studies were rated as high quality (7-8 stars) and four as moderate quality (5-6 stars); no study was rated as low quality. Points were most frequently lost in the comparability domain, reflecting variable adjustment for confounders such as patient age and graft diameter, and in the selection domain, reflecting the predominantly single-center, retrospective designs. The single RCT (Li et al. [37]) was judged to have some concerns using the RoB 2 tool, primarily due to limited reporting of allocation concealment. Overall, the body of evidence was of moderate quality, and these limitations should be considered when interpreting the pooled estimates.

**Table 2:** Risk of bias and methodological quality assessment of included studies Studies scoring 7-9 points on the NOS were considered high quality, 4-6 points were considered moderate quality, and 0-3 points were considered low quality. The randomized controlled trial was assessed separately using the RoB 2 tool. NOS, Newcastle-Ottawa scale; RoB 2, Cochrane risk of bias 2 tool

Study	NOS score	Quality assessment
Adams, 2023 [15]	8	High quality
Burrus, 2015 [17]	7	High quality
Darnley, 2016 [38]	6	Moderate quality
Heyworth, 2021 [20]	8	High quality
Jacobs, 2017 [18]	8	High quality
Kraeutler, 2018 [12]	7	High quality
Leo, 2016 [10]	6	Moderate quality
Li, 2015 [37]	N/A	Some concerns (RoB 2)
Mirzayan, 2023 [21]	8	High quality
Pennock, 2017 [19]	7	High quality
Perkins, 2019 [22]	8	High quality
Rao, 2021 [34]	7	High quality
Wang, 2018 [23]	6	Moderate quality
Xu, 2017 [24]	7	High quality
Xu, 2018 [35]	7	High quality
Yang, 2025 [36]	5	Moderate quality
Zheng, 2019 [14]	8	High quality

Graft failure

**Figure 2:**
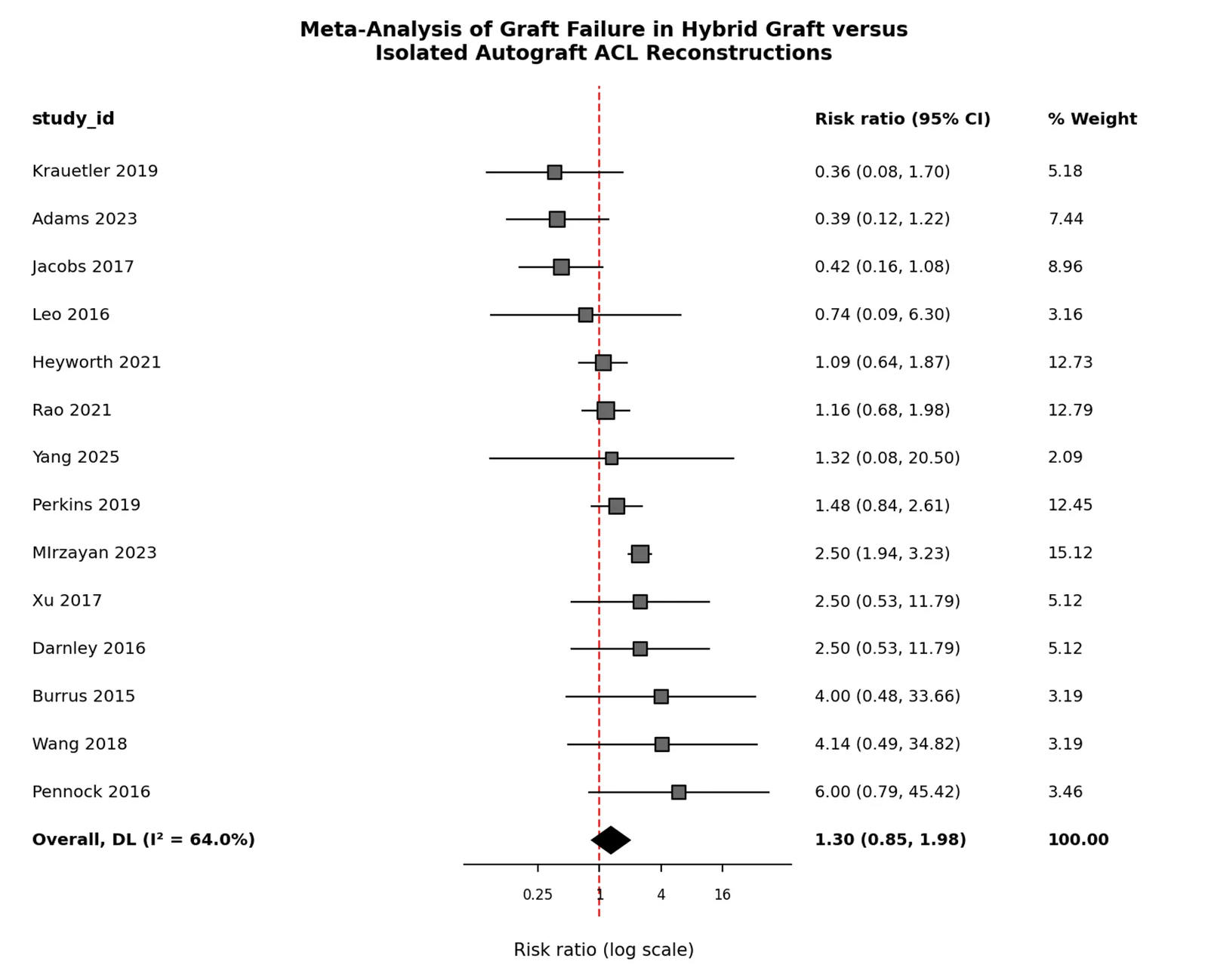
Meta-analysis of graft failure in hybrid graft versus isolated autograft ACL reconstructions ACL, anterior cruciate ligament

Fourteen (82.4%) of the 17 studies compared graft failure between the two cohorts [10,12,15,17-24,34,36,38]. Using a DerSimonian-Laird random-effects model, the pooled relative risk of failure was higher in the hybrid graft population (RR 1.30, 95% CI 0.85-1.98), but this difference was not statistically significant (p=0.23). Heterogeneity across studies was substantial (I²=64.0%, τ²=0.29; Q=36.07, df=13, p=0.0006) (Figure 2).

Patient-reported outcome scores

Thirteen (76.5%) of the 17 studies described validated PROMs, allowing objective comparison between graft types. The most commonly reported measures were the Lysholm knee score [14,17,19,23,35-37], IKDC score [10,12,14,17,23,35-38], and Tegner activity scale [14,19,23,35-37]. Meta-analysis was performed for each outcome.

**Figure 3:**
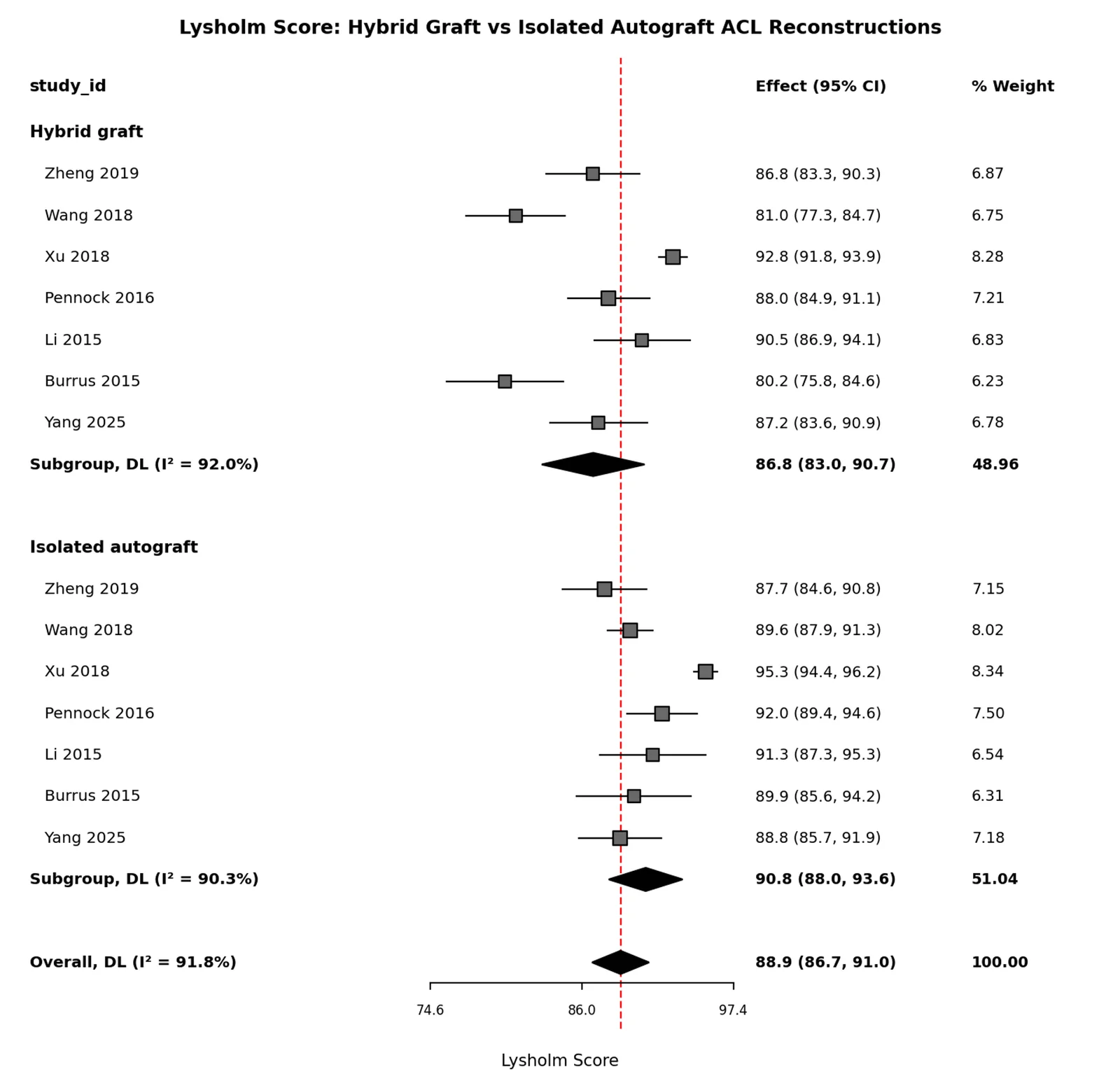
Meta-analysis of Lysholm scores in hybrid graft versus autograft ACL reconstructions ACL, anterior cruciate ligament

Seven studies reported Lysholm scores [14,17,19,23,35-37]. Using a random-effects model, the pooled Lysholm score was higher in the isolated autograft group by a WMD of 3.77 points (95% CI 1.47-6.07), and this difference was statistically significant (p=0.0013). Heterogeneity was moderate (I²=58.3%, τ²=5.05). Despite reaching statistical significance, the observed difference did not exceed the reported MCID of 10.6-11.1 points and is therefore unlikely to represent a clinically meaningful difference between graft types (Figure 3).

**Figure 4:**
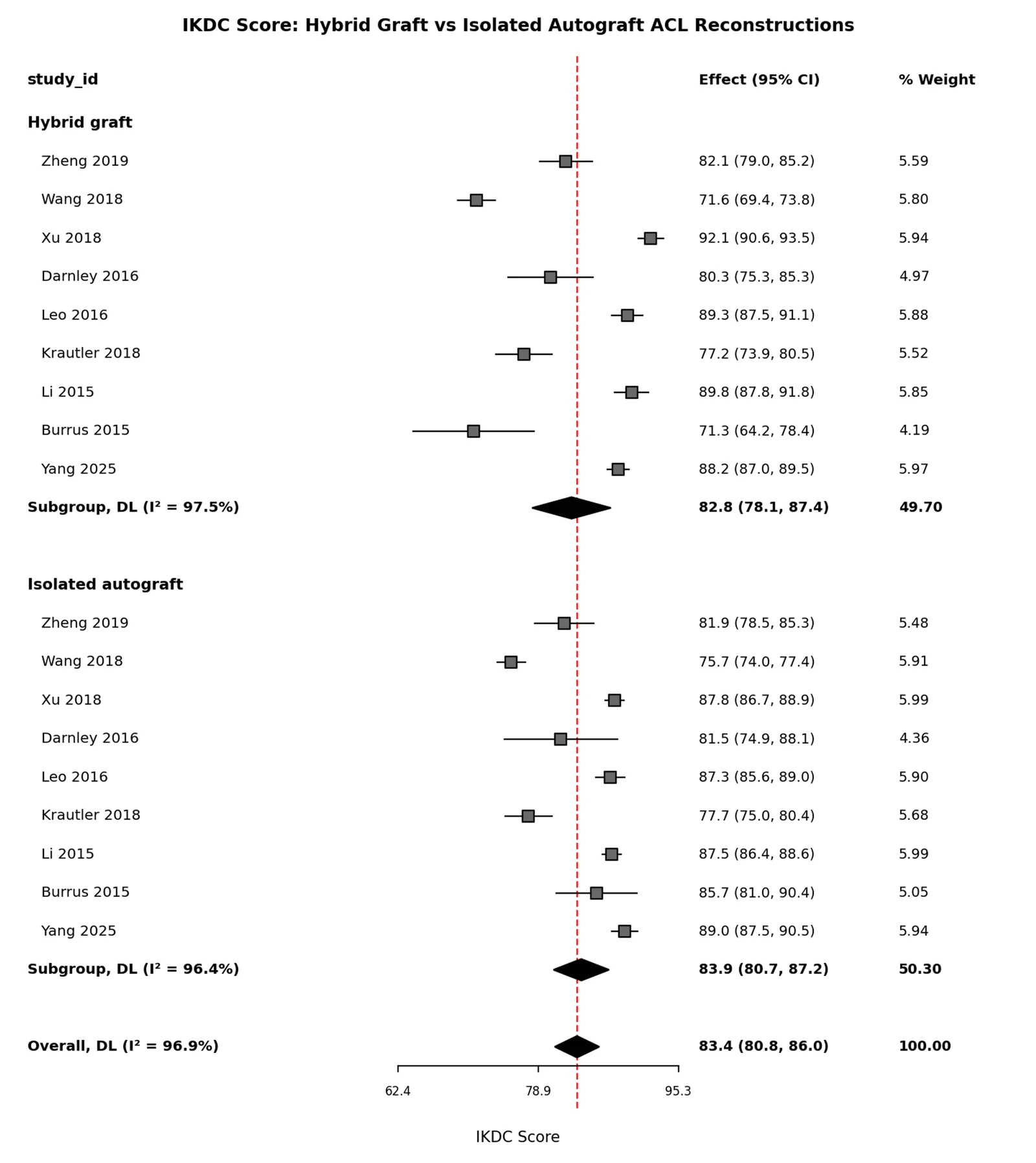
Meta-analysis of IKDC scores in hybrid graft versus autograft ACL reconstructions ACL, anterior cruciate ligament; IKDC, International Knee Documentation Committee

Nine studies reported IKDC scores [10,12,14,17,23,35-38]. Using a random-effects model, the pooled IKDC scores were similar between the isolated autograft and hybrid graft groups (WMD -0.31 favoring hybrid, 95% CI -2.72 to 2.11), with no statistically significant difference (p=0.80). Heterogeneity was high (I²=81.6%, τ²=9.68). This difference did not approach the reported MCID range of 6-17 points (Figure 4).

**Figure 5:**
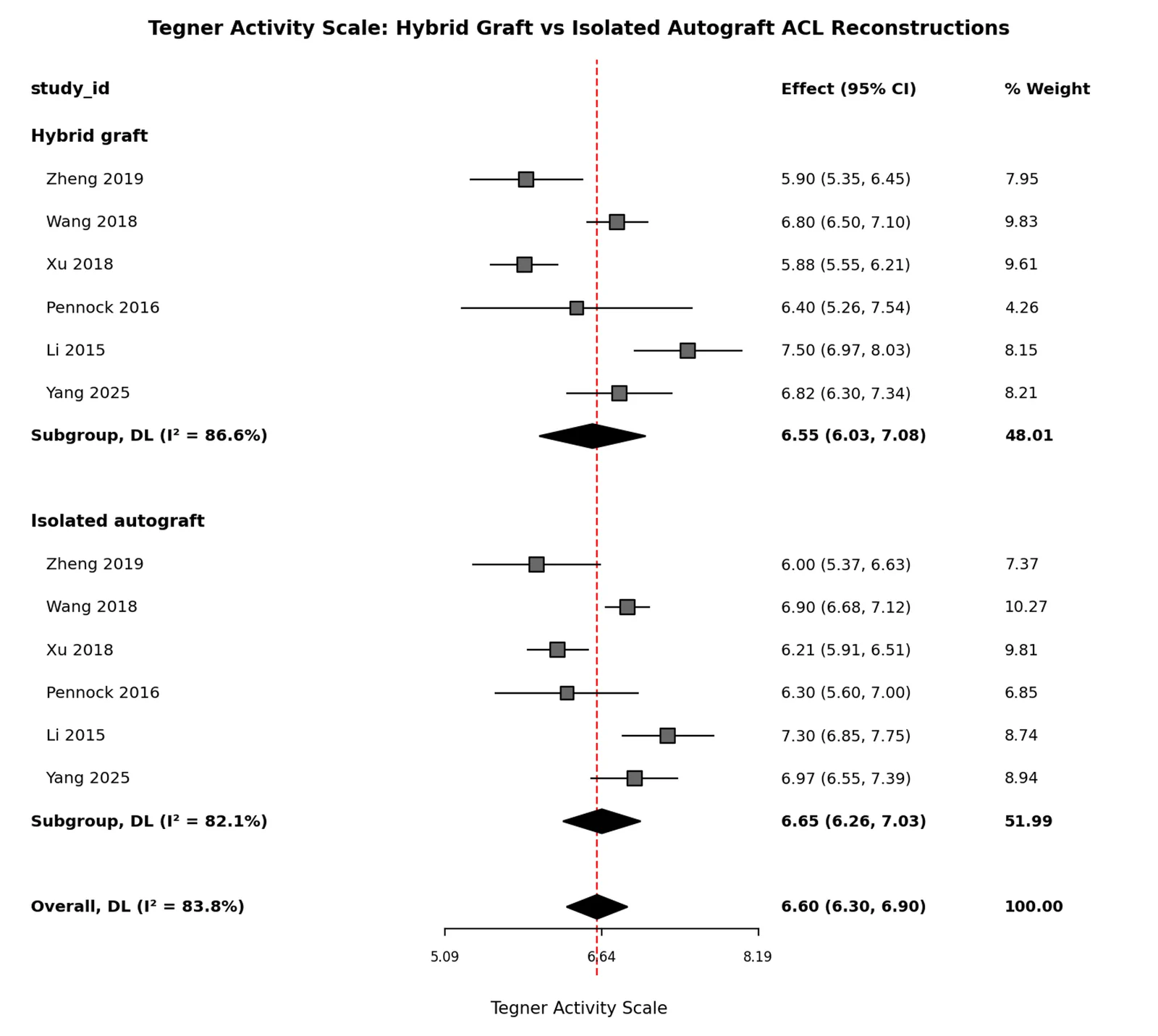
Meta-analysis of Tegner scores in hybrid graft versus autograft ACL reconstructions ACL, anterior cruciate ligament

Six studies reported Tegner activity scale scores [14,19,23,35-37]. Using a random-effects model, the pooled Tegner score was comparable between the isolated autograft and hybrid graft groups (WMD -0.13, 95% CI -0.36 to 0.10), with no statistically significant difference (p=0.28). Heterogeneity was negligible (I²=0%). This difference of approximately 0.1 points on the 10-point scale is not clinically meaningful (Figure 5).

Taken together, the pooled analyses showed no statistically or clinically meaningful advantage for either graft across any outcome. Table 3 summarizes the pooled estimates, heterogeneity, and interpretation for graft failure and the three PROMs. Across all four outcomes, the isolated autograft group scored marginally higher on the patient-reported measures and had a slightly lower pooled failure risk, but in every case, the difference fell short of statistical significance and, for the clinical scores, remained well below the established MCID.

**Table 3:** Summary of meta-analysis outcomes comparing hybrid graft versus isolated autograft ACL reconstruction Hybrid and autograft pooled columns reflect independent random-effects pooling of each arm's raw scores and are provided for descriptive purposes; the effect estimate (WMD) is calculated directly from each study's paired within-study difference and is the basis for the reported significance test. CI, confidence interval; IKDC, International Knee Documentation Committee; MCID, minimal clinically important difference; RR, risk ratio; WMD, weighted mean difference

Outcome	No of studies	Hybrid (pooled, 95% CI)	Autograft (pooled, 95% CI)	Effect estimate	I² (%)	Interpretation
Graft failure	14	-	-	RR 1.30 (0.85-1.98)	64	No significant difference (p=0.23)
Lysholm score	7	86.83 (82.96-90.70)	90.78 (88.00-93.56)	WMD 3.77 (1.47-6.07)	58.3	Statistically significant (p=0.0013) favoring autograft, but below the established MCID
IKDC score	9	82.76 (78.14-87.39)	83.91 (80.65-87.17)	WMD 0.31 (-2.11-2.72)	81.6	No statistically or clinically meaningful difference (p=0.80)
Tegner activity score	6	6.55 (6.03-7.08)	6.65 (6.26-7.03)	WMD 0.13 (-0.10-0.36)	0	No statistically significant difference (p=0.28)

Publication bias

Funnel plot inspection and Egger's regression test showed no statistically significant evidence of small-study effects or publication bias for graft failure (intercept p=0.22) or any patient-reported outcome (Lysholm p=0.41; IKDC p=0.13; Tegner p=0.38). However, three of the four outcomes included fewer than 10 studies, which limits the power of Egger's test to detect genuine asymmetry; these results should therefore be interpreted as reassuring but not conclusive.

Discussion

This systematic review and meta-analysis found no significant differences in patient-reported outcomes or graft failure rates between hybrid grafts and isolated autografts following ACLR. No clinically meaningful differences were identified across Lysholm, IKDC, or Tegner scores, and pooled graft failure rates were comparable between the groups.

Hybrid grafts are most commonly used when the harvested autograft is considered too small for reliable reconstruction. Although a smaller graft diameter has been associated with increased revision risk, the threshold at which augmentation becomes beneficial remains unclear. Published studies have used graft diameter cutoffs ranging from 7 mm to 8.5 mm, limiting direct comparison across cohorts. The available evidence suggests that hybrid augmentation increases graft diameter but does not always translate into superior clinical outcomes or lower failure rates.

Several individual studies have reported favorable outcomes with hybrid grafts, particularly when compared with smaller hamstring autografts. Jacobs et al. [18] and Kraeutler et al. [12] observed improved outcomes in selected cohorts with larger final graft diameters after augmentation. These findings support the theoretical benefit of increasing graft size; however, the observed advantages have not been consistently reproduced across the broader literature.

Conversely, multiple studies have reported either no benefit or inferior outcomes with hybrid grafts. Burrus et al. [17] and Perkins et al. [22] reported higher failure rates in hybrid graft cohorts, while Wang et al. [23] and Xu et al. [24,35] reported worse functional outcome scores in some comparisons. Importantly, these findings were not uniform across studies and were often influenced by differences in graft size, patient selection, and study design. When pooled in meta-analysis, these individual findings did not translate into statistically significant differences in clinical outcomes or graft failure rates.

Alternative and adjunct therapies

Several alternative strategies have been proposed for the management of small-diameter autografts. Preoperative magnetic resonance imaging-based assessment of hamstring tendon size has been investigated as a method to identify patients at risk for inadequate graft diameter and guide surgical planning [39]. Additionally, registry studies have demonstrated comparable revision risk between hybrid grafts and alternative graft constructs, emphasizing the importance of individualized graft selection [40]. Biologic augmentation strategies, including bone marrow aspirate concentrate (BMAC) [41], mesenchymal stem cell-based therapies [42], additional BMAC augmentation techniques [43], and platelet-rich plasma [44], have been proposed to enhance graft incorporation and biologic healing. Biocomposite scaffold technologies, such as BioBrace, have been recently introduced as adjunctive options for graft augmentation and differ mechanistically from the biologic augmentation strategies above in that they provide a collagen-based structural scaffold rather than a cellular or growth factor-based biologic supplement [13,45]. Although these approaches may offer theoretical advantages, current evidence remains limited and heterogeneous. Further prospective studies are needed to clarify their role in ACLR.

Strengths

This study has several strengths. The search strategy spanned three major databases with a predefined, reproducible Boolean search string, and study selection and data extraction were performed independently by at least two reviewers, with discrepancies resolved by a board-certified sports medicine orthopedic surgeon. Risk of bias was formally assessed for every included study using validated tools (NOS for cohort studies and RoB 2 for the included RCT), and the overall body of evidence was of moderate-to-high methodological quality, with no study rated as low quality. Pooled estimates were generated using random-effects models appropriate to the substantial heterogeneity observed, and publication bias was formally assessed via funnel plot and Egger's test for each outcome.

Limitations

Most included studies were retrospective cohort studies and, therefore, susceptible to selection bias and residual confounding despite generally favorable methodological quality scores. Furthermore, due to a compressed timeline, the study was not prospectively registered on PROSPERO. Heterogeneity existed with respect to graft diameter thresholds, augmentation techniques, graft failure definitions, and outcome reporting. These factors limited direct comparisons across studies and may have influenced pooled estimates. Further high-quality randomized trials can better define the role of hybrid graft augmentation in ACLR.

## Conclusions

Graft selection is one of several factors that may influence outcomes after ACLR. At the individual study level, both superior and inferior data regarding hybrid graft performance have been reported. However, the comprehensive literature review and meta-analysis in this study indicate that there are no clinically significant differences in ACLR failure rates or PROMs between hybrid-augmented and isolated autograft ACLR.
